# Sequential Organ Failure Assessment (SOFA) Score and Mortality Prediction in Patients With Severe Respiratory Distress Secondary to COVID-19

**DOI:** 10.7759/cureus.26911

**Published:** 2022-07-16

**Authors:** Mohamed Fayed, Nimesh Patel, Santhalakshmi Angappan, Katherine Nowak, Felipe Vasconcelos Torres, Donald H Penning, Anoop K Chhina

**Affiliations:** 1 Anesthesiology, Pain Management and Perioperative Medicine, Henry Ford Health System, Detroit, USA; 2 Anesthesia, Henry Ford Health System, Detroit, USA; 3 Research, Henry Ford Health System, Detroit, USA; 4 Anesthesiology and Perioperative Medicine, Duke University Health System, Durham, USA; 5 Anesthesiology, Henry Ford Health System, Detroit, USA; 6 Anesthesiology/Critical Care, Henry Ford Health System, Detroit, USA

**Keywords:** invasive mechanical ventilation, resource allocation, health care outcomes, resource-limited setting, severe respiratory failure, receiver operating characteristic (roc) analysis, prognostic modelling, predicted mortality, sofa score, covid 19

## Abstract

Background

This study looks at the validity of the sequential organ failure assessment score (SOFA) in detecting mortality in patients with Coronavirus disease of 2019 (COVID-19) pneumonia. Also, it is looking to determine the optimal SOFA score that will discriminate between mortality and survival.

Methods

It is a retrospective chart review of the patients admitted to Henry Ford Hospital from March 2020 to December 2020 with COVID-19 pneumonia who developed severe respiratory distress. We collected the following information; patient demographics (age, sex, body mass index), co-morbidities (history of diabetes mellitus, chronic kidney disease, chronic obstructive pulmonary disease, coronary artery disease, or cancer), SOFA scores (the ratio of arterial oxygen tension (PaO_2_) to the fraction of inspired oxygen, Glasgow Coma Scale (GCS) score, mean arterial pressure, serum creatinine level, bilirubin level, and platelet count) as well as inpatient mortality.

Results

There were 320 patients; out of these, 111 were intubated. The receiver operating characteristic (ROC) curve for SOFA at the moment of inclusion in the study had an area under the curve of 0.883. The optimal point for discrimination between mortality and survival is SOFA of 5. A SOFA score of less than two is associated with 100% survival, while a score of more than 11 is associated with 100% mortality.

Conclusions

SOFA score in COVID-19 patients with severe respiratory distress strongly correlates with the initial SOFA score. It is a valuable tool for predicting mortality in COVID-19 patients.

## Introduction

In 1996, Vincent et al. introduced the SOFA scoring system that was initially designed to sequentially assess the severity of organ dysfunction in patients who were critically ill from sepsis [1]. It is a scoring tool to evaluate organ dysfunction, using six organ system reproducible variables that measure disease severity during an intensive care unit (ICU) stay [2]. Since the early 1990s, the SOFA score has become an integrated tool to predict mortality in patients with multi-organ failure in the ICU [3,4]. Hence is being widely employed in critical care units worldwide for monitoring acute morbidity. Meanwhile, the COVID-19 pandemic has imposed a deleterious impact on the health systems worldwide, with an excessive burden across the intensive care units regarding exhaustion of resources, triaging, and physician burnout. Therefore, there has been a need to validate the available disease severity scoring systems like the SOFA score or other scoring systems like Acute Physiology and Chronic Health Evaluation (APACHE) [5]. This validation will eventually help triage patients, translating to meaningful utilization of the medical resources systematically. However, there are some concerns that the studied populations on both could not represent the behavior of novel or emerging infectious diseases. Indeed, the concern showed to be true early on in the H1N1 pandemic, with patients showing higher survivability than expected by SOFA scores [6,7]. The COVID-19 seemed to bring findings opposite, with a higher than average ICU mortality [8,9].

The pathology of COVID-19 involves multiple organ systems; however, the respiratory system takes the main brunt, resulting in pneumonia and acute respiratory failure. Respiratory system failure is the leading cause of death in these patients [10], and intubation, including surgical airway, could also be challenging [11]. Using a variety of outcome-predicting scoring systems, like the SOFA score, is needed as a reliable prognostic indicator for critically ill patients [3]. Nevertheless, considerable literature has not been on assessing the accuracy of the conventional SOFA score. Some evidence favored the SOFA score as a predictive test, and other shreds of evidence have different opinions [12,13].

Furthermore, there has not been any literature assessing the validity of the SOFA score from the onset of severe respiratory distress in patients with COVID-19 pneumonia. We presented our novel research on the SOFA score in patients with COVID-19; we used time zero of the onset of severe respiratory distress and looked at the worst SOFA score within 48 hours. This research can help physicians predict mortality and outcomes to support decision-making, bed utilization, and maximum life savings per ICU bed. Also, this research will help communicate with family and other relevant teams, including palliative care and end-of-life discussions.

## Materials and methods

The institutional review board reviewed and approved the study. The procedures were followed per the ethical standards of the responsible committee on human experimentation and the Helsinki Declaration of 1975. This research was appropriate in design and met the Federal Guidelines requirements, 45 CFR Part 46 and 21 CFR Part 50. The board name is Edsel Board; the approval number is 14370. The approval date is November 5, 2020, study tile Impact of intubation time vs. non-intubation in patients with moderate to severe acute respiratory distress syndrome secondary to COVID-19 pneumonia. Informed consent was waived by the regulatory board. We identified eligible patients using the Henry Ford Health System EPIC electronic medical record database. We retrospectively reviewed the medical records of all patients admitted to the Henry Ford Hospital in Detroit, Michigan, a level 1 trauma tertiary care center between March 13, 2020, and December 12, 2020. Eligible patients were diagnosed with COVID-19 pneumonia confirmed with a positive polymerase chain reaction test (nasal swab) and developed severe respiratory distress. We defined severe respiratory distress as COVID-19 pneumonia with bilateral infiltrates indicated by chest x-ray and at least one of the following criteria: (1) respiratory rate more than 30 for at least two hours or (2) oxygen saturation less than 93% for at least two hours. We excluded patients who had a “do not intubate” or a “do not resuscitate” order at enrollment from the analysis. Variables collected included patient demographics: age, sex, body mass index (BMI)(expressed as the ratio kg/m^2^), co-morbidities such as coronary artery disease (CAD), chronic obstructive pulmonary disease (COPD), diabetes mellitus (DM), chronic kidney disease (CKD), and history of cancer. Hospital SOFA scores (calculated by collecting required variables - the ratio of PaO_2_ to the fraction of inspired oxygen, GCS) score, mean arterial pressure, use of vasopressors, serum creatinine level, bilirubin level, and platelet count). We collected the worst values of the SOFA score observed within 48 hours of meeting the criteria of severe respiratory distress, and we looked into in-hospital mortality. 

The numeric variables were summarized using mean and interquartile range (IQR). The data analysis was performed, and plots were created using GraphPad Prism 5.0, SPSS 20, and built-in and custom routines in MATLAB (MathWorks, Inc). For receiver operating characteristic (ROC) curves calculation, we used publicly available JavaScript programs JROCFIT and JLABROC4 (Eng J. ROC analysis: web-based calculator for ROC curves. Baltimore: Johns Hopkins University [updated March 19, 2014; cited April 3, 2022]. Available from: http://www.jrocfit.org). The output was subsequently exported and treated in MATLAB. For linear trend analysis, we grouped the patients according to intervals of SOFA Score. P-values ≤ 0.05 were considered statistically significant in all analyses. We expressed the results as mean ± standard deviation.

## Results

**Figure 1:**
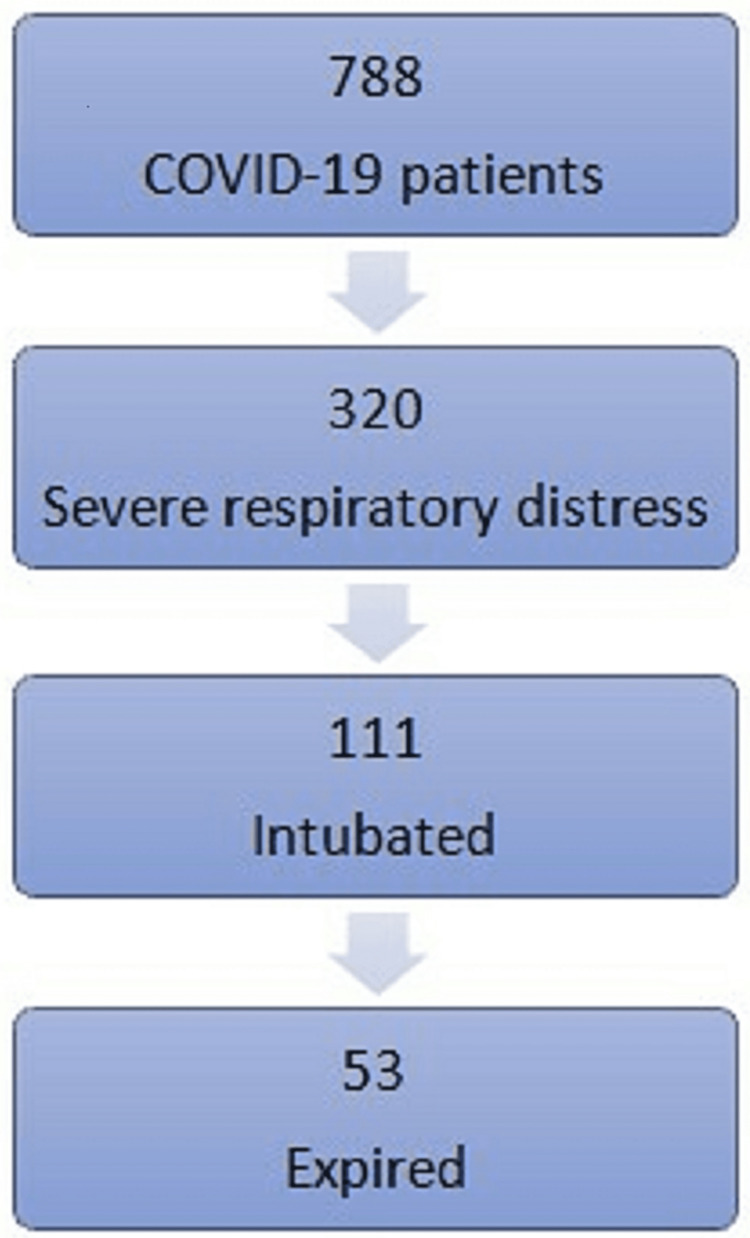
Patient recruitment in our facility.

We found 788 patients with confirmed COVID-19 patients admitted to our facility during this period. Of whom, 320 patients met the eligibility criteria for severe respiratory distress. One hundred eleven patients received intubation and mechanical ventilation. Fifty-three of the intubated patients died, there were 17 mortality cases in non-intubated patients (Figure 1).

The mean age of the 320 included patients was 62 years old with an IQR of 53-74 years; males accounted for 51%. Mean BMI was 33 with IQR 27-38. The most common co-morbidity was chronic kidney disease (47%); this was followed by diabetes mellitus 46% (159) of patients (Table 1).

**Table 1:** Patients’ demographics and history of co-morbidities. IQR: Interquartile range, M: male, n: number.

Parameter	Value
Age mean (IQR)	62 years (53-74)
Sex (M) n (%)	163 (51%)
Body mass index, mean (IQR)	33 (27-38)
Coronary artery disease n (%)	25 (7%)
Diabetes mellitus n (%)	159 (46%)
Chronic obstructive pulmonary disease n (%)	44 (13%)
Chronic kidney disease n (%)	159 (47%)
Cancer n (%)	43 (12.6%)

Table 2 shows the number of patients and associated mortality in grouped SOFA scores and the incidence of mortality in each group in the original SOFA score. A SOFA score from 0 to 1 is associated with 100% survival, while a SOFA score greater than 11 is associated with 100% mortality (Table 2).

**Table 2:** Number of patients with associated mortality in grouped SOFA scores. SOFA: Sequential Organ Failure Assessment, n: number.

Grouped SOFA Scores	COVID patients, n	COVID mortality, n (%)	Predicted mortality (%) according to the original SOFA score
0-1	81	0 (0%)	0
2-3	71	4 (5.6%)	6.4%
4-5	67	14 (20.9%)	20.2%
6-7	51	18 (35.3%)	21.5%
8-9	26	13 (50%)	33.3%
10-11	17	14 (82.3%)	50%
12-14	6	6 (100)	95.2%
>14	1	1 (100%)	95.2%
Number Total	320	70	

**Figure 2:**
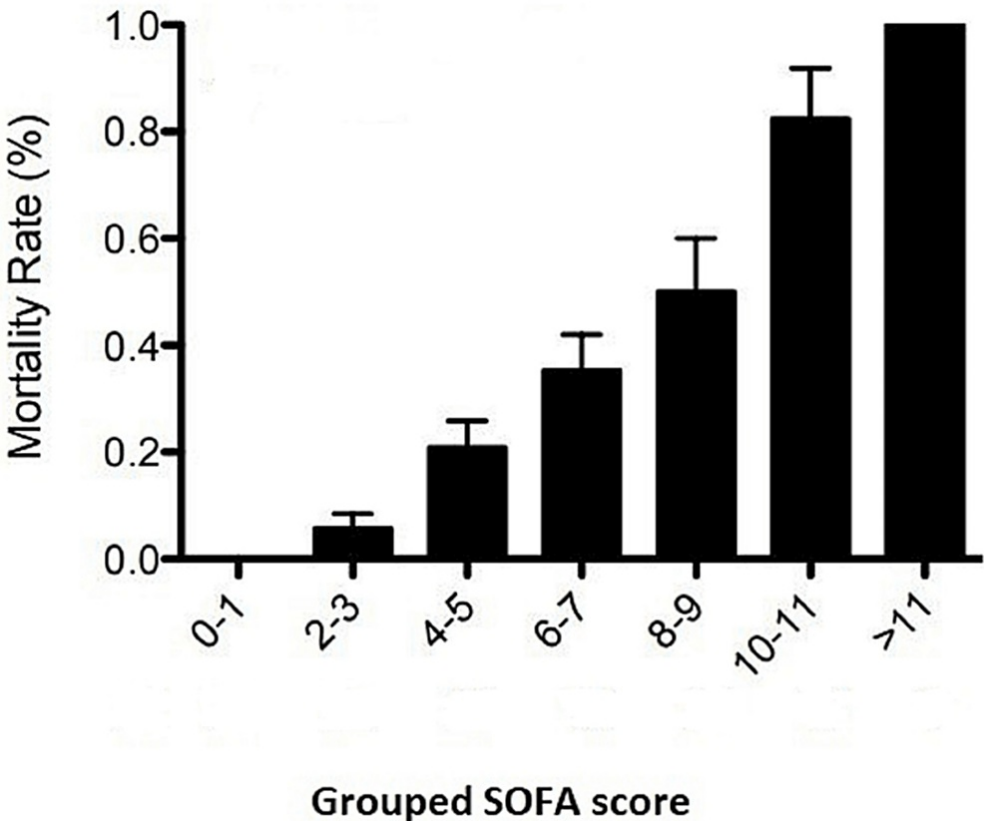
Mortality rate in various grouped SOFA scores. SOFA: Sequential Organ Failure Assessment

Incidence of mortality increases in SOFA scores between 6 and 11 compared to the original SOFA score. SOFA score 6-7 is associated with 35% mortality compared to 20% in the original SOFA score. Figure 2 shows the mortality rate for grouped SOFA Scores. Linear trend analysis showed a statistically significant positive correlation with a p-value < 0.001 (Figure 2).

**Figure 3:**
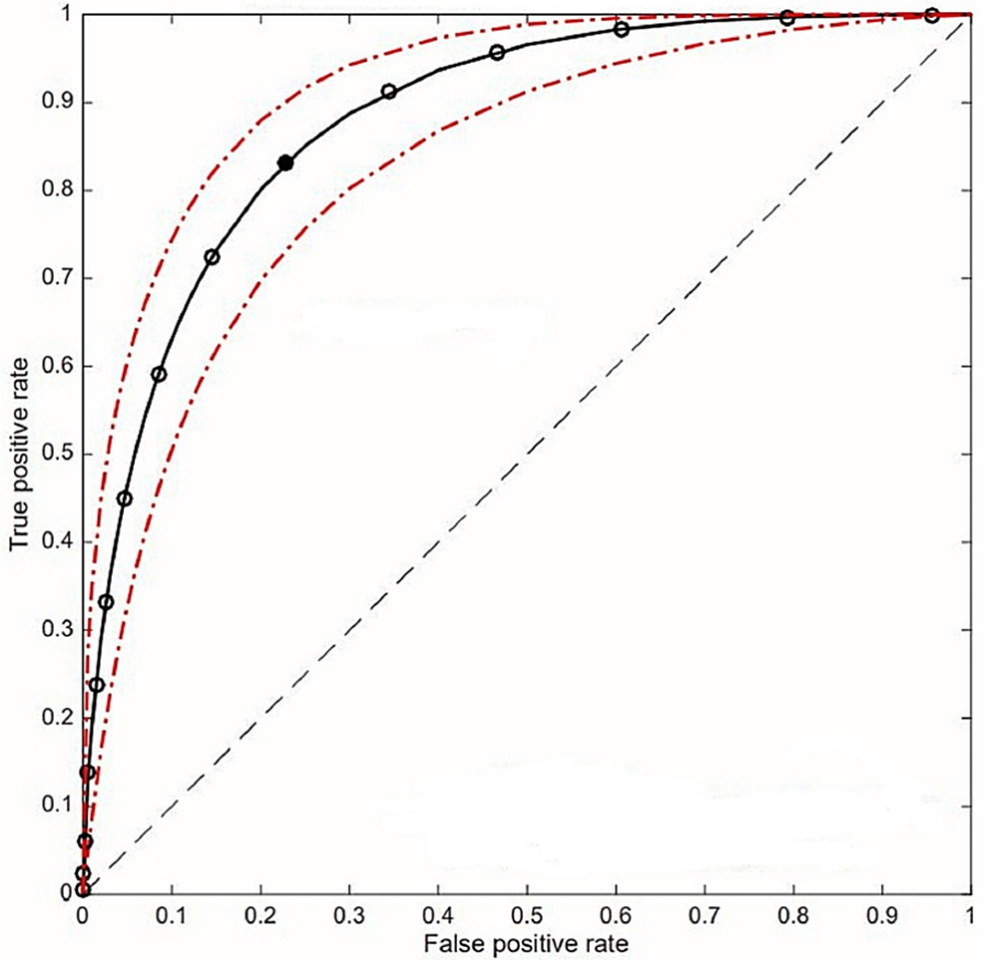
SOFA score's ROC in predicting mortality. ROC: Receiver operator curve, SOFA: Sequential Organ Failure Assessment, continuous black line: fitted ROC curve, interrupted red line: 95% confidence interval of the fitted ROC, white circles: SOFA score corresponding points, black circle: optimal discriminating point (SOFA score = 5)

We constructed the Receiver operator curve (ROC) to evaluate the predictive value of the SOFA score to predict mortality in COVID-19 patients with severe respiratory distress. Figure 3 shows the ROC curve of various SOFA scores at the onset of severe respiratory distress diagnosis (and inclusion in the study). The area under the curve (AUC) was used to evaluate the diagnostic accuracy of the SOFA score. ROC showed an area under the curve (AUC) of 0.883, which indicates a good test. The optimal point for discrimination (best balance between false positive fraction and true positive fraction) corresponds to SOFA equal to 5 (the point nearest to the left upper corner of the graph). This point has a sensitivity and specificity to detect 74 and 80% mortality, respectively (Figure 3).

## Discussion

The initial impression was that the SOFA score would be an invalid tool to predict mortality in COVID-19 patients. Compared to the multisystem scoring tool in SOFA scores, these patients usually suffered from single organ failure, namely the respiratory system. However, with the evolving detailed description of the spectrum of COVID-19 disease, involvement of the cardiovascular, coagulation system, and central nervous system has been well proven. COVID-19 can induce the activation of the complement and coagulation system, which is related to disease severity [14-16].

The ROC curve displays the trade-off between sensitivity or true positive rate (TPR) and specificity (1 - False positive rate [FPR]). Classifiers that give curves closer to the left upper corner, the better test will discriminate between true positive (mortality) and false positive (survival). As a baseline, a random classifier is expected to give points lying along the diagonal (FPR = TPR). In addition, the area under the ROC curve gives an idea about the benefit of using the test(s) in question. Hence, the higher the AUC, the better the test's performance distinguishing between true positive and false positive. ROC curves are used clinically to choose the most appropriate cut-off for a test. ROCs are valid if their AUC is greater than 0.5, and an AUC greater than 0.7 is considered acceptable, while greater than 0.8 is considered good. Our study showed AUC for SOFA predicting mortality in patients with severe respiratory distress from COVID-19 pneumonia was 0.883, which indicates the appropriateness of using the SOFA score to predict mortality in this cohort of patients. A cut-off value of 5 for SOFA score and a sensitivity and specificity of 74% and 80%, respectively. This result suggests that a SOFA score ≥5 can predict the severity and mortality in patients with COVID-19 pneumonia.

Interpreting the SOFA score depends on whether the physician wants to use it as a diagnostic, prognostic, or resource allocation tool. This prognostication will help physicians gauge the severity and possible outcomes, which can guide resource allocation, triaging patients, and facilitating end-of-life discussions with the family. In our study, patients with a SOFA score of 0-1 at admission and within the first 48 hours had 100% survival, while scores more than 11 had 100% mortality rates. A score of 5 is the cut-off between mortality and false positive (survival) rates. During the pandemic with limited resources, this is the point where maximum resource utilization occurs; more lives will be saved per bed in the ICU when compared to higher scores. For example, for ten beds in ICU with a SOFA score of 8, we will be able to rescue two patients compared to a SOFA score of 5, where we will be able to rescue eight patients.

Few studies looked at SOFA scores in COVID-19 patients. So far, no one has looked at the SOFA score with the onset of severe respiratory distress as a prognostic value in mortality. A study looked at 367 patients with the initiation of high flow nasal cannula as a clinically significant time point. They found AUC 0.766, and they found that SOFA score perform better than the APACHE score [17]. We believe taking initiation of HFNC as a surrogate for ICU admission is very non-specific, and in fact, two-thirds of their patient populations were not admitted to ICU. Another study looked at patients admitted to ICU in Belgium; they used time zero of ICU admission regardless of the clinical state of the patients. This study was done during the peak of the pandemic. SOFA score was higher in these patients, and they did not find SOFA score as a good predictor of mortality [9], this could be explained by limited understanding of COVID-19 as well as limited resources during the peak of the pandemic. A retrospective study published in 2021 analyzed the prognostic value of SOFA score in 117 patients with COVID-19 pneumonia [18]. Their study illustrated that the SOFA score could be used to evaluate the severity and 60-day mortality of COVID-19; they found an AUC of 0.9. However, one of the significant drawbacks of this study is that they included all patients with COVID-19 at random time points and did not include time zero from admission. In addition to this, the study was from a developing country with a different Healthcare system. Their results cannot be extrapolated to this population cohort in the United States. Another study published in 2021 put forward that the SOFA score possesses inadequate discriminant accuracy for ventilator triage of COVID-19 patients. However, they retrospectively analyzed only intubated patients when admitted to the ICU; this might skew the AUC for the SOFA score [9]. Ultimately, our study confirmed that the SOFA scoring for COVID-19 pneumonia had a good correlation with the conventional SOFA scoring system for sepsis patients admitted to the ICU.

Limitations

Our study is limited by its retrospective design. It is also a single-center study, hence has its known shortcomings. In addition, this outcome might not reflect in other parts of the United States, developed countries, or developing ones. There are ongoing advancements in treating COVID-19, including antivirals, monoclonal antibodies, and various vaccines, but most of them prevent progression to severe disease. SOFA score does not consider the age of the patient, which might play a role in other treatment modalities, for example, lung transplantation.

Our study focused on patients with severe respiratory distress; however, populations may vary in disease severity between hospitals. However, our center is a tertiary center with a high-volume unit, so our data may be a valuable resource to support and kindle further research on this cohort.

## Conclusions

In conclusion, severity scoring systems like SOFA have the potential to be used as a good tool for predicting mortality in COVID-19 pneumonia patients. Furthermore, combining these scores with other clinical elements and imaging may help stratify the severity and risk of death from COVID-19 pneumonia. Therefore, the inclusion of these tools in decision strategies could provide early detection of high-risk groups, avoid delayed medical attention, facilitate resource allocation and improve clinical outcomes in COVID-19 pneumonia patients.
